# Potential Risk of Local Transmission of Mpox in Nepal: An Emerging Public Health Concern

**DOI:** 10.1002/hsr2.71168

**Published:** 2025-09-12

**Authors:** Yogendra Shah, Ankit Kumar Singh, Bipin Kumar Acharya, Mandira Lamichhane, Ram Singh Dhami, Kishor Pandey, Shyam Prakash Dumre, Kouichi Morita, Basu Dev Pandey, Meghnath Dhimal

**Affiliations:** ^1^ Planetary Health Research Centre Kathmandu Nepal; ^2^ Central Department of Zoology Tribhuvan University Kathmandu Nepal; ^3^ National One Health Alliance for Nepal Kathmandu Nepal; ^4^ Central Department of Microbiology Tribhuvan University Kathmandu Nepal; ^5^ Institute of Tropical Medicine Nagasaki University Nagasaki Japan; ^6^ DEJIMA Infectious Disease Research Alliance Nagasaki University Nagasaki Japan; ^7^ Nepal Health Research Council Kathmandu Nepal

**Keywords:** monkeypox, Nepal, outbreak, transmission, zoonotic

## Abstract

**Background and Aims:**

Monkeypox (Mpox) is a zoonotic disease caused by the monkeypox virus, a double‐stranded DNA virus of the *Orthopoxvirus* genus with two major clades (Clades I and II), with Clade IIb responsible for most global cases reported during 2022–2024. By July 2024, more than 100,000 Mpox cases and 200 deaths were reported worldwide. Nepal has recently confirmed three imported Mpox cases, raising concerns about the potential for local transmission amid increased labor migration and peacekeeping troops abroad. This study aims to trace out the emerging threat and risk of Mpox transmission in Nepal and preparedness and prevention strategies based on global experiences.

**Methods:**

We reviewed global and national epidemiological reports and Mpox case data from the government authority of Nepal to identify Mpox clades with Clade II at the National Public Health Laboratory (NPHL).

**Results:**

In Nepal, three cases have been confirmed, all linked with recent travel history to the Middle East country (Saudi Arabia). Mpox reported in Nepal belonged to Clade IIa by sequencing. Additionally, the risk of Mpox transmission among Nepalese individuals in the UN peacekeeping forces stationed in Africa, including a few endemic countries, may be elevated due to shared living conditions, as human‐to‐human transmission can occur through direct contact, fomites, or respiratory secretions.

**Conclusion:**

Nepal should strengthen molecular laboratories, enforce national guidelines, enhance border health monitoring, raise public awareness, and ensure timely diagnosis and treatment. Coordinated strategies and preparedness are crucial to prevent potential outbreaks and enhance national readiness against future Mpox threats in Nepal. It is recommended that the Government of Nepal adopt and implement a One Health approach for the early detection, control, and prevention of Mpox to minimize transmission risks.

## 1 Introduction

Mpox, previously known as monkeypox, is a zoonotic viral disease caused by the monkeypox virus, an enveloped double‐stranded DNA virus belonging to the *Orthopoxvirus* genus within the *Poxviridae* family. The virus is classified into two distinct clades: Clade I (Subclades Ia and IIb) and Clade II. Most of the Mpox outbreaks reported worldwide in 2022–2023 were caused by the Clade IIb strain. The global Mpox outbreak has resulted in over 100,000 confirmed cases and 200 deaths worldwide linked with IB as of July 31, 2024 [1]. The mpox was first reported from Denmark in 1959, and it has been endemic in West and Central African countries since 1970 [2]. Later, the cases of mpox were reported from other countries [2]. On August 14, 2024, the WHO declared a Public Health Emergency of International Concern (PHEIC) due to the rapid spread of the new Clade 1B strain, particularly in the eastern Democratic Republic of Congo (DRC), and surrounding Ethiopia and Burundi. Its recent detection in regions such as Sweden and Thailand highlights the increasing global threat. The common symptoms of Mpox include skin rashes or mucosal lesions, fever, headache, muscle aches, backache, fatigue, and lymphadenopathy. The incubation period typically ranges from 7 to 14 days, but it can extend up to 21 days in some cases. The transmission route of Mpox is close contact with infected persons or animals through contaminated objects [1, 3, 4]. Mpox is closely associated with HIV infection, presenting significant challenges as 50% of cases are diagnosed in children in Africa, and women are more frequently affected than men, particularly in regions with poor sanitation and shared living spaces, and also, females are primary caregivers, clade variation, and other reasons. However, a study conducted by Coutinho et al. showed that males were more affected than females, mainly cis‐gender by Mpox [5]. Also, the previous study highlights that a strong association between Mpox and HIV; conversely, it was specified that not only HIV but also sexually transmitted infections could be associated with Mpox: syphilis, anal high‐risk human papillomavirus infections, and infections by *Neisseria gonorrhoea* and *Mycoplasma* [6]. Furthermore, the limited availability of CD4 and viral load testing facilities for HIV patients in low‐ and middle‐income countries, such as Nepal, heightens the risk due to the strong association between Mpox and HIV [7].

A 9‐year‐old boy was the first person who was diagnosed with Mpox in 1970 in the DRC. Since then, the disease has been reported in many African nations, including Burundi, Cameroon, the Central African Republic (CAR), Côte d'Ivoire, Gabon, Liberia, Kenya, Nigeria, Rwanda, South Africa, and Uganda [1]. There are two strains of MPXV Clades I and II that cause Mpox. Among them, some cases of Clade II have been reported from Thailand, Sweden, and Nepal (Clade II A.2) based on sequencing results from the National Public Health Laboratory (NPHL), Ministry of Health and Population, Nepal [8], while Clade I cases have been reported from Africa [1]. In fact, there are a huge number of UN peacekeeping personnel (Nepal Army, Nepal Police, and Armed Police Force) stationed in African nations, including CAR, and Congo, which are known for sustained Mpox transmission. Nepal is the second‐largest contributor of UN peacekeeping forces (consistently ranking among the top troop‐contributing countries) and has been sending troops for more than 6 decades now. Therefore, the risk of Mpox importation and local transmission progressing to an outbreak is always there in Nepal.

In 2024, Africa reported over 20,000 cases and more than 600 deaths. Notable outbreaks have occurred from 2022 to 2024 among 120 countries, with over 70 cases reported in the United States in September 2018, and subsequent outbreaks in the United Kingdom (2018–2022), Singapore (2018), and the United States again from July to November 2021. According to Promedmail.org, confirmed cases were also reported in 2022 in Spain (14 cases), France (1 case), Belgium (2 cases), Germany (1 case), Italy (1 case), Sweden (1 case), Portugal (14 cases), the United Kingdom (9 cases), Canada (2 cases), and Australia (1 case), with a case fatality ratio of approximately 3%–6% [7, 9]. On December 18, 2024, two Mpox cases involving individuals returning from the United Arab Emirates were verified in Kerala, India, a neighboring country. There are presently no ongoing instances in India, which has reported 33 cases since 2022 (17 in Kerala and 16 in Delhi) [8, 9]. Furthermore, throughout India, thorough national guidelines for managing Mpox have been put into place [8, 9]. According to Nepal's Epidemiology Disease Control Division (EDCD), a 44‐year‐old migrant from Sindhuli district who returned and travel history from Saudi Arabia tested positive for Mpox on December 29, 2024. In June 2023, a 60‐year‐old foreign national who often visits Nepal and other nations was the subject of Nepal's first Mpox case [10, 11]. The second case was also confirmed as Mpox in a 36‐year‐old man from the Tanahun district, who had recently returned from Saudi Arabia. The patient was admitted to the isolation ward of the Sukraraj Tropical and Infectious Disease Hospital after the NPHL confirmed the case. The patient's condition remains stable now [12, 13]. Given that over 170,000 Nepalese traveled abroad for work in the first quarter of the fiscal year 2024/25—primarily to the UAE, Malaysia, and Saudi Arabia—the high risk of Mpox transmission is heightened due to frequent international travel and labor migration [11, 12, 13, 14, 15]. Additionally, the third case of Mpox was also confirmed in Nepal, which is a public health concern due to potential spread of the virus in the future (Table 1). Therefore, the risk of Mpox transmission among Nepalese in the Middle East may be elevated due to shared living conditions, as human‐to‐human transmission can occur via direct contact, fomites, or respiratory secretions [16].

**Table 1:** Mpox cases confirmed in Nepalese migrants with a travel history from Saudi Arabia.

Mpox cases	Age/sex	District	Travel history	Sequencing result	Confirmed date/organization	Clade
First	60/F	Kathmandu^a^	Saudi Arabia	—	June 30, 2023/NPHL	—
Second	36/M	Tanahun	Saudi Arabia	hMpxV	December 20, 2024/NPHL	IIa
Third	44/M	Sindhuli	Saudi Arabia	hMpxV	December 29, 2024/NPHL	IIa

*Source:* Monkeypox (Mpox) sequencing result from National Public Health Laboratory (NPHL), Kathmandu, Nepal (https://nphl.gov.np/page?id=135) [8].

^a^ Foreigner.

*Recommendations and future action plan*. A One Health approach is crucial for detecting new DNA and RNA viruses. It involves active surveillance, isolation of suspected human and animal cases, laboratory testing for zoonotic and infectious diseases, and providing early warnings to public health authorities [17, 18, 19]. It is suggested that the Government of Nepal could consider the following recommendation:

- *Strengthen laboratory capacities*. Expand and upgrade molecular laboratories established during the COVID‐19 pandemic in each province to ensure rapid and molecular diagnosis by automated nucleic acid extraction and real‐time polymerase chain reaction of outbreak diseases like Mpox.
- *Strengthen international health desks*. To stop the spread of viruses, place health monitoring desks at international borders, transit hubs, and airports. Follow international health regulation (IHR) guidelines.
- *Development and implement national guidelines*. Develop comprehensive practical guidelines to control and manage Mpox outbreaks across the country. For instance, neighbouring country India has made a similar one. Laboratory testing of suspected and probable cases for early detection and identify clades, using similar laboratory and clinical surveillance techniques.
- Establishing treatment facilities for the treatment including painful rashes, coinfection like Kaposi sarcoma, HIV‐positive cases.
- Develop an AI model with a web application to analyze uploaded skin images and determine the suspected Mpox patients at the only International airport settings in Nepal.
- *Policy and research support*. Using a One Health approach, update research findings for planners and policymakers to enhance DNA viral infection diagnosis, genotyping tools, and treatment plans.
- *Public awareness and reporting*. Utilize various media platforms to continuously raise awareness about Mpox symptoms, prevention, and control. Encourage timely reporting through the Ministry of Health and Population hotline (1115) to ensure prompt action. Surveillance: Implement community‐based surveillance, along with monitoring wastewater and environmental samples.
- *Vaccination*. Consider vaccination as a strategy for postexposure prophylaxis.

## 2 Future Directions

**Figure 1:**
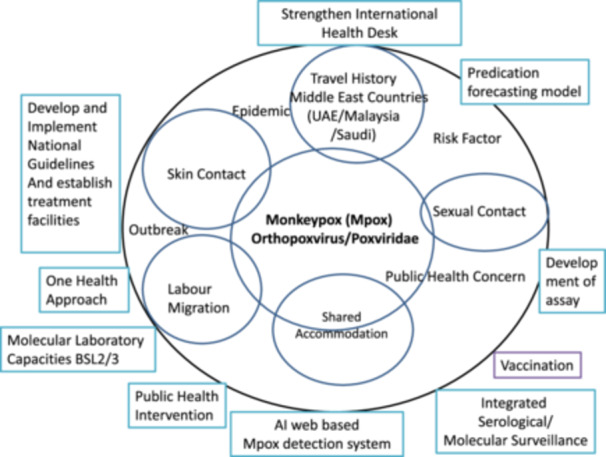
Framework of Mpox for future direction.

Animal reservoirs, including rodents and monkeys, pose the constant risk of outbreaks of Mpox in Nepal. Due to weak healthcare infrastructure, poor sanitation, sharing close spaces, and low public awareness also exacerbate these risks in Nepal. Strong collaborative efforts among the government, healthcare professionals, and the public are crucial to enhancing surveillance and preparedness for Mpox. Furthermore, health professionals must educate patients, while the government should allocate resources for coordinated responses. On the other hand, public adherence to hygiene practices and timely medical attention for suspected cases can further mitigate risks. Therefore, a farsighted and comprehensive approach, as well as cutting‐edge like development of an AI model with a web application to determine the suspected Mpox patients at international airport settings in Nepal, is essential for Nepal to reduce risk factors of Mpox transmission and build potency against future public health challenges (Figure 1).

## Author Contributions

**Yogendra Shah:** conceptualization, methodology, writing – original draft, writing – review and editing. **Ankit Kumar Singh:** conceptualization, methodology, writing – original draft, writing – review and editing. **Bipin Kumar Acharya:** conceptualization, methodology, writing – review and editing, writing – original draft. **Mandira Lamichhane:** conceptualization, methodology, writing – original draft, writing – review and editing. **Ram Singh Dhami:** software, writing – review and editing. **Kishor Pandey:** conceptualization, methodology, supervision, writing – original draft, writing – review and editing. **Shyam Prakash Dumre:** conceptualization, methodology, supervision, writing – original draft, writing – review and editing. **Kouichi Morita:** supervision, writing – review and editing, conceptualization. **Basu Dev Pandey:** conceptualization, writing – review and editing, supervision. **Meghnath Dhimal:** conceptualization, writing – review and editing, supervision.

## Conflicts of Interest

The authors declare no conflicts of interest.

## Transparency Statement

The lead authors, Yogendra Shah, Basu Dev Pandey, and Meghnath Dhimal, affirm that this manuscript is an honest, accurate, and transparent account of the study being reported; that no important aspects of the study have been omitted; and that any discrepancies from the study as planned (and, if relevant, registered) have been explained.

## Data Availability

The data were derived from the following resources available in the public domain:
− National Public Health Laboratory, https://nphl.gov.np/page?id=135
−
*The Kathmandu Post*, https://kathmandupost.com/health/2024/12/21/nepal-confirms-second-case-of-monkeypox
−
*The Kathmandu Post*, https://kathmandupost.com/health/2024/12/31/nepal-confirms-third-mpox-case-experts-warn-of-possible-spread
−
*The Kathmandu Post*, https://kathmandupost.com/health/2024/12/28/nepal-to-send-mpox-sample-to-who-lab-in-thailand-for-clade-analysis
